# Genetic analysis of posterior medial barrel subfield (PMBSF) size in somatosensory cortex (SI) in recombinant inbred strains of mice

**DOI:** 10.1186/1471-2202-9-3

**Published:** 2008-01-07

**Authors:** Taha A Jan, Lu Lu, Cheng-Xiang Li, Robert W Williams, Robert S Waters

**Affiliations:** 1 Department of Anatomy and Neurobiology, University of Tennessee Health Science Center, Memphis, Tennessee 38163, USA

## Abstract

**Background:**

Quantitative trait locus (QTL) mapping is an important tool for identifying potential candidate genes linked to complex traits. QTL mapping has been used to identify genes associated with cytoarchitecture, cell number, brain size, and brain volume. Previously, QTL mapping was utilized to examine variation of barrel field size in the somatosensory cortex in a limited number of recombinant inbred (RI) strains of mice. In order to further elucidate the underlying natural variation in mouse primary somatosensory cortex, we measured the size of the posterior medial barrel subfield (PMBSF), associated with the representation of the large mystacial vibrissae, in an expanded sample set that included 42 BXD RI strains, two parental strains (C57BL/6J and DBA/2J), and one F1 strain (B6D2F1). Cytochrome oxidase labeling was used to visualize barrels within the PMBSF.

**Results:**

We observed a 33% difference between the largest and smallest BXD RI strains with continuous variation in-between. Using QTL linkage analysis from WebQTL, we generated linkage maps of raw total PMBSF and brain weight adjusted total PMBSF areas. After removing the effects of brain weight, we detected a suggestive QTL (likelihood ratio statistic [LRS]: 14.20) on the proximal arm of chromosome 4. Candidate genes under the suggestive QTL peak for PMBSF area were selected based on the number of single nucleotide polymorphisms (SNPs) present and the biological relevance of each gene. Among the candidate genes are *Car8 *and *Rab2*. More importantly, mRNA expression profiles obtained using GeneNetwork indicated a strong correlation between total PMBSF area and two genes (*Adcy1 *and *Gap43*) known to be important in mouse cortex development. GAP43 has been shown to be critical during neurodevelopment of the somatosensory cortex, while knockout *Adcy1 *mice have disrupted barrel field patterns.

**Conclusion:**

We detected a novel suggestive QTL on chromosome 4 that is linked to PMBSF size. The present study is an important step towards identifying genes underlying the size and possible development of cortical structures.

## Background

The remarkable amount of normal variability in the architecture of the central nervous system (CNS) has a substantial genetic component composed, in part, of multiple polymorphic genes each of which has a small effect on phenotypic variation. Quantitative trait locus (QTL) mapping is an efficient means of associating positions in the genome with variation in a phenotype [1]. Such quantitative genetic approaches applied in recombinant inbred (RI) strains of mice have been used to map and characterize genes responsible for heritable variation in a number of CNS morphologic phenotypes including ventricular size [2], hippocampal structure, size, and cell number [3,4], and cerebellar size and structure [5]. RI strains possess several advantages in QTL analysis. Firstly, RI strains are fully inbred and multiple individuals can be phenotyped to derive an accurate strain average. Secondly, many of the RI strains have been densely genotyped, eliminating this often costly and time-consuming step.

Recently, quantitative genetic methods were applied to characterize the heritability of cortex characteristics in inbred and RI strains of mice [6,7]. Li and colleagues began examining the underlying genetic basis of variability in the size of barrel subfields of the primary somatosensory (SI) cortex in adult mice [8]. Woolsey and Van der Loos [9] first described the organization of cell aggregates, called barrels, in layer IV of the mouse SI and showed that these barrels represent the vibrissae and sinus hairs on the contralateral face [10]. These barrels are organized in a continuous manner from the posterior medial region of SI, designated as the posterior medial barrel subfield (PMBSF), associated with the representation of the large mystacial vibrissae, to the anterior regions designated as the anterior lateral barrel subfield (ALBSF) associated with the representation of the sinus hairs on the face and snout. Barrels increase in number while decreasing in size from the PMBSF to the ALBSF. Further studies led to the characterization of additional barrel fields associated with forepaw and hindpaw representations [11-14].

Li and colleagues took advantage of the barrel model by quantitatively comparing the cortical representation of the vibrissae representation, specifically the combined areas of the PMBSF/ALBSF, between common inbred strains and between 10 BXD RI strains [8]. To generate these RI strains, two progenitor strains, C57BL/6J and DBA/2J, were intercrossed and subsequent generations were inbred. The resulting panels of inbred strains have fixed homozygous genotypes at each locus, with parental (C57BL/6J and DBA/2J) alleles segregated among the strains [15]. In addition to finding significant variation in PMBSF/ALBSF area, suggesting that this phenotype is a polygenic trait, we reported two markers on chromosomes 8 and 10 that were suggestively linked to total PMBSF/ALBSF area in 10 BXD strains. However, it was unknown how robust these results would be when additional strains were phenotyped and measurements were restricted to the well-defined PMBSF area. The present work addresses these questions by examining the PMBSF area in 42 strains of BXD mice. Because PMBSF/ALBSF area was previously shown to be highly heritable [8], we focused on maximizing the number of strains. In this study, we detected a suggestive QTL on the proximal arm of chromosome 4; to date, this is the first QTL associated with normal variation in PMBSF area. We further identified carbonic anhydrase related protein 8 (*Car8*) as a potential candidate gene that may modulate PMBSF area variability.

## Results

### PMBSF organization

**Figure 1:**
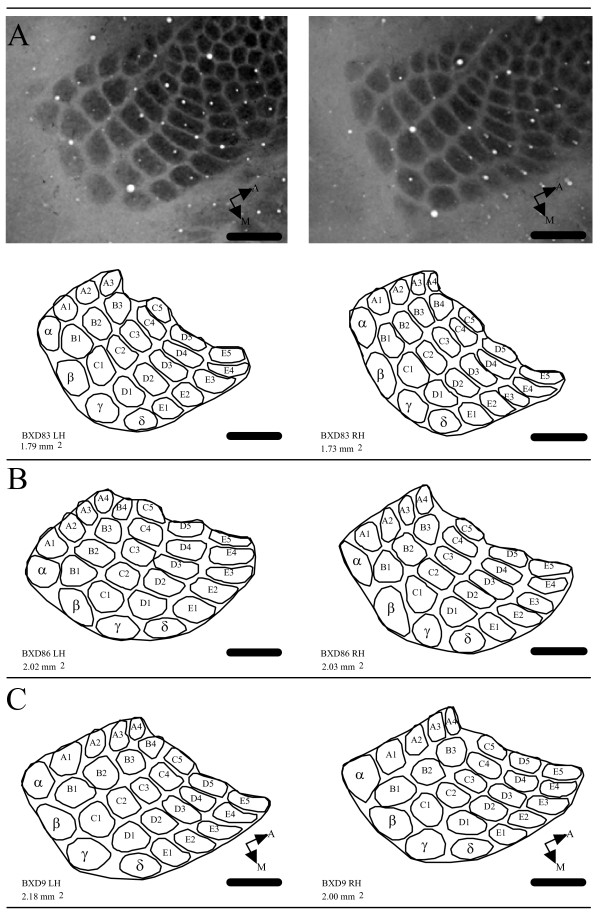
Examples of hemispheres with asymmetrical number of barrels from three BXD mice. (A) Photomicrograph and outline drawings of left (LH) and right (RH) hemispheres from one BXD83 mouse. Total PMBSF areas are indicated below line drawings. Number of barrels does not significantly affect total PMBSF area. (B-C) Line drawings of PMBSF barrel representations from two additional BXD mice (BXD86, BXD9). Scale 500 μm.

The PMBSF was examined in CO stained tissue in 140 mice from 45 strains (BXD = 42, parentals = 2, F1 = 1) (Table 1). The PMBSF consists of five well-defined anterior-posterior running rows (A row through E row) each representing one large mystacial vibrissae on the contralateral face. Rows A and B typically contain four barrels, while rows C-E contain five barrels. Four straddler barrels (α, β, γ, δ) form the posterior border of the PMBSF. Together, a total of 27 barrels form the PMBSF and the area defining these barrels was measured in the present study. It is worth noting that in a number of cases (n = 22) one barrel from either the A or B or both rows were found missing in one or both hemispheres, but the general overall PMBSF pattern was not disrupted. Furthermore, there was no significant difference (p = 0.86) between total PMBSF areas of hemispheres with asymmetrical number of barrels. Several examples of the PMBSF area are shown in Figure 1.

**Table 1:** Age, body weight, brain weight, PMBSF area, and adjusted PMBSF area values

Group	Total Cases (n = 10)	Age (days)	Body Wt (g)	Brain Wt (mg)	PMBSF Area (mm^2^)	Adj PMBSF Area (mm^2^)
C57BL/6J	7 (1)	44.10 ± 0.40	16.67 ± 0.95	415.57 ± 4.73	2.00 ± 0.03	1.96 ± 0.03
DBA/2J	6 (5)	43.00 ± 0.63	16.52 ± 0.99	345.83 ± 7.57	2.08 ± 0.06	2.14 ± 0.06
B6D2F1/J	5 (2)	48.80 ± 0.49	22.04 ± 0.91	423.62 ± 3.71	2.50 ± 0.07	2.45 ± 0.13
BXD1	3 (2)	45.00 ± 3.00	16.81 ± 2.55	394.00 ± 31.72	1.70 ± 0.05	1.68 ± 0.07
BXD2	2 (1)	45.00 ± 0.00	22.05 ± 1.99	375.45 ± 5.65	1.81 ± 0.09	1.82 ± 0.14
BXD9	2 (1)	43.00 ± 0.00	18.26 ± 1.44	403.90 ± 23.50	1.96 ± 0.13	1.93 ± 0.22
BXD12	2 (1)	42.00 ± 0.00	16.80 ± 2.15	397.50 ± 3.30	1.89 ± 0.13	1.87 ± 0.18
BXD24	4 (3)	46.00 ± 0.00	18.41 ± 1.47	357.50 ± 13.69	2.00 ± 0.10	2.03 ± 0.15
BXD25	4 (4)	42.00 ± 0.00	15.14 ± 0.50	361.00 ± 3.44	1.97 ± 0.03	2.00 ± 0.09
BXD31	7 (5)	43.43 ± 0.37	16.65 ± 1.05	363.21 ± 10.60	1.76 ± 0.05	1.78 ± 0.10
BXD32	4 (2)	42.75 ± 1.70	20.30 ± 1.19	380.48 ± 14.42	1.82 ± 0.06	1.83 ± 0.10
BXD33	3 (3)	52.00 ± 0.00	21.15 ± 0.27	350.90 ± 3.37	1.90 ± 0.05	1.94 ± 0.11
BXD39	2 (1)	45.00 ± 0.00	20.59 ± 1.83	352.30 ± 14.40	1.73 ± 0.07	1.77 ± 0.11
BXD40	2 (1)	47.00 ± 0.00	18.80 ± 1.06	428.40 ± 4.10	2.02 ± 0.07	1.96 ± 0.12
BXD43	2 (1)	42.00 ± 0.00	18.45 ± 1.50	399.55 ± 33.35	2.38 ± 0.03	2.35 ± 0.08
BXD44	2 (1)	46.50 ± 1.50	18.50 ± 1.48	422.85 ± 0.35	1.82 ± 0.07	1.76 ± 0.13
BXD51	2 (1)	45.50 ± 3.50	19.56 ± 2.07	420.60 ± 21.90	2.15 ± 0.03	2.10 ± 0.06
BXD55	2 (1)	49.50 ± 2.50	19.43 ± 2.74	445.85 ± 12.25	2.25 ± 0.10	2.16 ± 0.14
BXD56	2 (1)	47.00 ± 0.00	20.80 ± 0.03	388.60 ± 6.00	1.92 ± 0.12	1.91 ± 0.17
BXD60	2 (1)	44.00 ± 0.00	20.92 ± 1.87	405.85 ± 1.75	1.96 ± 0.06	1.93 ± 0.11
BXD61	2 (1)	44.00 ± 2.00	19.78 ± 2.71	421.35 ± 20.65	2.00 ± 0.02	1.94 ± 0.11
BXD62	1 (1)	44.00 ± 0.00	22.07 ± 0.00	388.20 ± 0.00	1.59 ± 0.00	1.58 ± 0.00
BXD63	3 (2)	48.67 ± 2.33	19.02 ± 1.42	375.90 ± 16.76	1.87 ± 0.06	1.88 ± 0.11
BXD64	3 (2)	47.67 ± 2.33	21.58 ± 2.39	336.17 ± 18.28	1.80 ± 0.04	1.86 ± 0.10
BXD66	4 (3)	48.25 ± 0.25	20.45 ± 1.61	311.73 ± 17.13	1.88 ± 0.02	1.98 ± 0.09
BXD68	2 (1)	42.00 ± 0.00	19.91 ± 1.86	400.65 ± 8.25	1.90 ± 0.08	1.87 ± 0.12
BXD69	2 (1)	44.00 ± 0.00	18.60 ± 1.00	415.30 ± 5.00	1.99 ± 0.01	1.94 ± 0.06
BXD70	4 (4)	42.00 ± 0.00	13.85 ± 1.03	349.65 ± 10.67	1.86 ± 0.05	1.90 ± 0.10
BXD71	2 (2)	44.50 ± 0.50	22.85 ± 0.08	455.90 ± 8.80	2.11 ± 0.08	2.01 ± 0.15
BXD72	5 (2)	49.00 ± 0.00	16.03 ± 0.57	385.80 ± 3.96	2.05 ± 0.07	2.04 ± 0.13
BXD73	6 (5)	51.83 ± 2.04	21.89 ± 1.44	432.70 ± 10.16	1.76 ± 0.02	1.69 ± 0.07
BXD75	3 (2)	42.67 ± 0.33	19.33 ± 0.17	394.60 ± 6.30	1.71 ± 0.03	1.69 ± 0.09
BXD77	3 (2)	43.00 ± 1.00	23.54 ± 1.07	413.97 ± 9.11	2.03 ± 0.05	1.98 ± 0.12
BXD80	4 (2)	53.50 ± 4.33	19.47 ± 2.23	380.38 ± 8.88	1.95 ± 0.11	1.95 ± 0.16
BXD83	2 (1)	44.00 ± 0.00	18.83 ± 2.04	421.55 ± 29.55	1.80 ± 0.04	1.75 ± 0.06
BXD84	2 (1)	51.00 ± 0.00	15.80 ± 0.51	370.40 ± 1.20	2.06 ± 0.09	2.08 ± 0.15
BXD86	2 (1)	49.00 ± 0.00	17.86 ± 2.66	446.45 ± 12.85	1.97 ± 0.05	1.88 ± 0.09
BXD88	4 (4)	51.50 ± 0.50	19.29 ± 1.21	389.03 ± 8.70	2.07 ± 0.05	2.06 ± 0.11
BXD89	2 (0)	42.00 ± 0.00	16.04 ± 1.19	346.35 ± 10.65	2.05 ± 0.06	2.09 ± 0.11
BXD90	7 (3)	53.43 ± 1.88	18.43 ± 1.52	382.03 ± 11.24	2.09 ± 0.08	2.09 ± 0.12
BXD92	2 (1)	45.00 ± 0.00	24.13 ± 4.84	450.55 ± 9.45	2.05 ± 0.03	1.95 ± 0.07
BXD93	4 (2)	55.00 ± 0.00	18.84 ± 0.60	388.40 ± 1.59	2.25 ± 0.04	2.25 ± 0.10
BXD96	2 (1)	43.00 ± 0.00	21.70 ± 2.28	474.55 ± 10.65	1.69 ± 0.01	1.56 ± 0.06
BXD97	2 (1)	42.00 ± 0.00	21.69 ± 2.00	417.05 ± 3.85	2.15 ± 0.01	2.10 ± 0.07
BXD98	2 (1)	40.00 ± 0.00	18.29 ± 1.87	368.45 ± 8.05	1.71 ± 0.01	1.72 ± 0.08

Values are given as average ± S.E.M

### Heritability

Broad-sense heritability (h^2^) of PMBSF area was calculated using raw data for all phenotyped cases. The computed value for PMBSF area variation was approximately 58% (h^2 ^= 0.58). This confirmed previous findings where similar heritability values (60%) were calculated for combined PMBSF/ALBSF using only 10 strains [8]. This similarity in heritability further substantiates our previous study that the use of a small number of animals per strain (n = 2.90 in this study) is appropriate for mapping purposes given a large number of phenotyped strains. As expected, PMBSF area adjusted for brain weight showed a lower heritability of approximately 41% (h^2 ^= 0.41).

### Brain weight and body weight

Brain and body weight values of all phenotyped cases correlate. Pearson's product-moment correlation showed that brain and body weights correlate at r = 0.44 (p < 0.0001). Group mean correlation of brain and body weights for all strains was r = 0.37 (p = 0.013).

### Parental strain differences

PMBSF areas of DBA/2J and C57BL/6J were 2.08 ± 0.06 mm^2 ^and 2.00 ± 0.03 mm^2^, respectively, making the DBA/2J larger by 4.24%. This difference was not statistically significant (p = 0.11). However, when the data were adjusted for brain weight, there was a significant (p = 0.008) difference between DBA/2J and C57BL/6J (2.14 ± 0.06 mm^2 ^and 1.96 ± 0.03 mm^2^, respectively) making the DBA/2J larger by 8.50%. Total brain weight of DBA/2J animals was significantly (p < 0.0001) smaller than that of C57BL/6J animals (0.35 ± 0.01 g, 0.42 ± 0.01 g respectively). In comparison, body weights of the two strains did not vary with DBA/2J = 16.53 ± 0.99 g and C57BL/6J = 16.67 ± 0.95 g.

### Sex and PMBSF area

The majority of strains used contained at least one male and one female; however, same sex mice were used in seven strains (see Table 1). Animal sex did not show a strong relationship to PMBSF area and thus was not used as a predictor of PMBSF area. Using a Pearson's product-moment correlation, sex correlated with PMBSF area (r = 0.03). A simple regression analysis confirmed the significance of this relationship where we failed to reject the null hypothesis. The regression also showed that sex only accounted for approximately 0.1% of the variance seen in PMBSF area (F_1,138 _= 0.15, p = 0.70).

### Age and PMBSF area

Animals used in this study fell within a small age range: 40 – 61 days old. Simple correlation analysis among all phenotyped individuals showed age to be moderately correlated with PMBSF area (r = 0.26, p = 0.0016). Likewise, age and PMBSF area of group means correlated, albeit with less significance (r = 0.26, p = 0.08). A simple regression analysis revealed that age accounts for approximately 7.0% of total PMBSF area variability (F_1,138 _= 10.4, p = 0.0016). However, this may be misleading given that age was also coincidently correlated with strain (r = 0.22, p = 0.0091). For this reason, age was excluded as a factor when modeling PMBSF area variability.

### Effects of body and brain weights on PMBSF area

Body weight correlated significantly with total PMBSF area in all individual cases at a modest level (r = 0.20, p = 0.02). Approximately 3.8% of variance in PMBSF area may be accounted for by body weight (F_1,138 _= 5.44). Similarly, brain weight also correlated significantly with PMBSF area (r = 0.25, p = 0.0034). However, unlike body weight, brain weight accounted for about 6.1% of the variance seen in PMBSF area (F_1,138 _= 8.89).

### PMBSF area variability and data modeling

Our results showed a 33% difference between PMBSF area means of the largest and smallest BXD RI strain measurements (see Table 1). We utilized direct correlations between PMBSF area and other variables to explore genetic relationships. A linear model was utilized to distinguish among the most significant factors for the prediction of PMBSF area variability.

Three factors were taken into consideration in the linear model: sex, body weight, and brain weight. Strain was excluded as a predictor because it is the genetic factor that we hypothesized to account for PMBSF area variability. Using all individual cases, we observed that sex was the least significant factor (p = 0.46) in predicting PMBSF area variability; sex was thus the first factor removed from the model. Next, body weight became the least significant factor (p = 0.24) and was therefore removed. Brain weight remained a significant factor both in the presence of sex and body weight and of course as the last remaining factor as well (p = 0.0034). This was consistent with the finding that brain weight accounts for approximately 5.4% of the variability in PMBSF area, the largest percentage of variance as predicted by other factors. We therefore adjusted PMBSF area for brain weight and used the adjusted values for QTL mapping.

### QTL modulating brain and body weights

**Figure 2:**
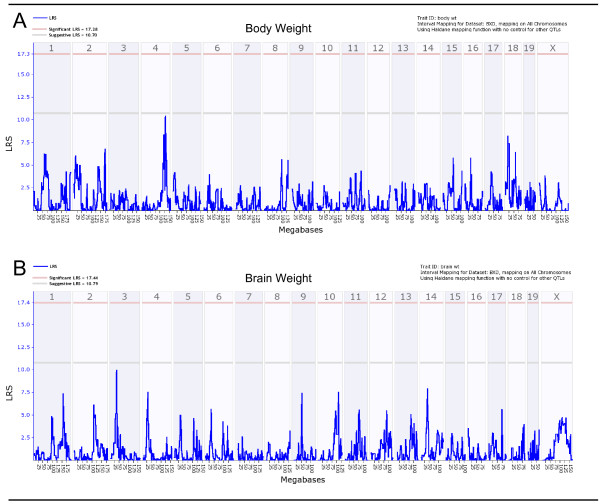
Genome-wide linkage maps of body and brain weights. Linkage maps of body weight (A) and brain weight (B) show no significant or suggestive peaks that overlap with total PMBSF area QTL. Lower gray horizontal line: suggestive LRS genome-wide threshold at p ≤ 0.63. Upper red horizontal line: significant LRS genome-wide threshold at p ≤ 0.05.

In this study, all QTL mapping was completed using only BXD RI strains (n = 42). To further confirm that our PMBSF area QTLs are not due to variations in brain and body weights, we mapped the QTLs for these traits on WebQTL using raw data. Figure 2A shows a QTL map of body weight using raw data. Clearly, this map does not show any significant QTLs. It should be noted however, that there was a signal near the suggestive threshold on chromosome 4, however the location is remote from the PBMSF area QTL as discussed below. The QTL map of brain weight using raw data is shown in Figure 2B. Again, only a couple of near-suggestive outcomes were observed with one on chromosome 4. None of the detected signals, including the one on chromosome 4 for brain weight, overlapped with our proposed PMBSF area suggestive QTL.

### QTL modulating PMBSF area

**Figure 3:**
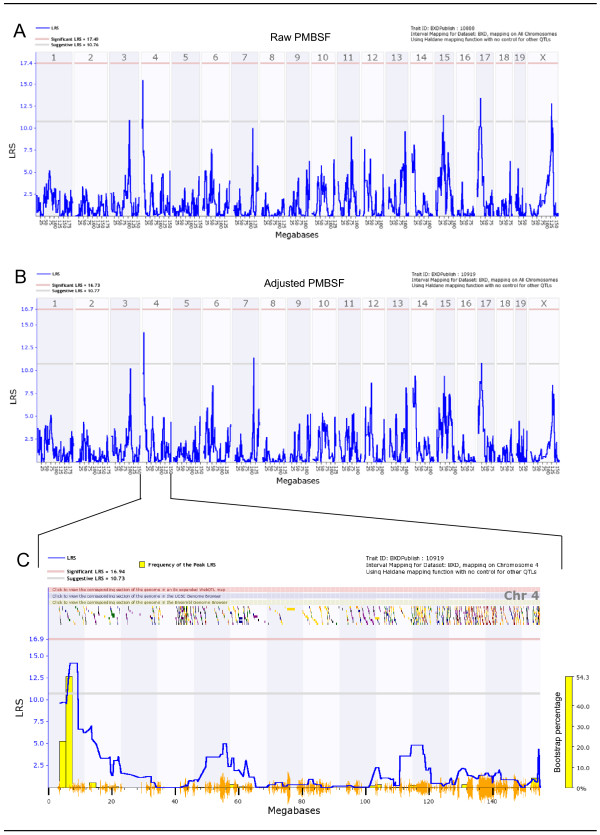
Genome-wide total PMBSF raw and adjusted linkage maps and chromosome 4 interval map. (A) Genome-wide linkage map of raw PMBSF area shows two suggestive QTLs on chromosomes 4 and 17. (B) Total PMBSF area linkage map after adjustment for brain weight shows a suggestive QTL on chromosome 4. Chromosome 17 linkage is not observed here. (C) Chromosome 4 interval map of adjusted total PMBSF area. Genes spanning the interval of 5.5 to 9.0 Mb on chromosome 4 were examined. Lower gray horizontal line: suggestive LRS genome-wide threshold at p ≤ 0.63. Upper red horizontal line: significant LRS genome-wide threshold at p ≤ 0.05. Yellow histogram: frequency of peak LRS (bootstrap analysis). Orange seismograph marks indicate SNP density.

We used our 42 BXD strains to map total PMBSF area QTL. Both raw data and adjusted values of PMBSF area were utilized for mapping in order to test whether our data modeling affected position of the identified PMBSF area QTL. By examining the simple regression QTL map of raw PMBSF area (Figure 3A), we immediately noticed two suggestive QTLs, one on the proximal arm of chromosome 4 and the other on chromosome 17. In addition, a couple of other signals (chromosomes 15 and X) were also observed to cross the suggestive threshold. When PMBSF area was adjusted for brain weight, the suggestive QTL on chromosome 4 remained largely unchanged, in terms of its significance and its location (Figure 3B). However, most of the other suggestive QTLs observed in the raw data map had diminished signals, in particular those on chromosomes 17 and X. Marker regression analysis using WebQTL revealed four loci with highly suggestive LRS values, all at 14.20 (suggestive threshold LRS = 10.68). The detected loci are: rs3674982, rs13477546, gnf04.004.855, rs13477551.

Interval regression mapping (Figure 3C) of chromosome 4 with 1000 permutations and bootstrap analysis shows a detailed view of the total PMBSF area suggestive QTL. Bootstrap analysis is a method of testing the reliability of the peak QTL signal by randomly producing a new sample from the original data set. The newly created sample set contains the same number of cases as the original set, however one or more of the cases may be duplicated and/or removed. The bootstrap analysis histogram at right in Figure 3C shows that the suggestive QTL is regenerated at the same location approximately 54% of the time, further suggesting the reliability of the signal within chromosome 4.

The QTL signal spans a relatively small region, from 5.5 to 9.0 Mb. Although there are no obvious multiple QTLs, we tested for interaction among different loci given that there are a number of suggestive loci. Both LRS Interaction and LRS full pair-scan analyses revealed no significant two-loci epistatic interactions.

### Candidate genes

We detected 9 genes that lie under the chromosome 4 interval of 5.5 to 9.0 Mb (Table 2). We utilized a 1.5-LOD support interval around the peak value of the suggestive QTL to derive the 5.5 to 9.0 Mb range. In order to increase the likelihood of detecting potential candidate genes, we first prioritized the genes by the number of SNPs present within each gene. Biological relevance was the next factor that went into consideration when identifying potential candidates. With these criteria in mind, we identified two genes of particular interest that are discussed below (*Car8 *and *Rab2*). *Car8 *is a *cis*-acting gene whereas *Rab2 *is a *trans*-acting gene.

**Table 2:** Candidate Genes Chr4 (5.5 Mb – 9.0 Mb)

Number	Gene Name	Start Location (Mb)	Gene Length (Mb)	Gene ID
1	1700012H17Rik	5.571325	155.766	242297
2	3110003A22Rik	6.118251	28.684	68053
3	Cyp7a1	6.192758	10.020	13122
4	Sdcbp	6.292862	29.937	53378
5	Nsmaf	6.323372	58.036	18201
6	Tox	6.614604	304.100	252838
7	Car8	8.068640	97.548	12319
8	Rab2	8.462790	72.059	59021
9	AK172025	8.617552	176.380	-

### Correlative analysis

Microarray data analysis is a powerful method that may be used in conjunction with QTL analysis to detect genes of interest that may be expressed in remote regions of the genome. We used RNA expression profiles of whole brain from one of the GeneNetwork databases (INIA Brain mRNA M430 (Jan06) PDNN) to detect genes of interest that are correlated with our adjusted PMBSF area phenotype. An initial correlation query resulted in the detection of 2000 transcripts from the entire genome that were correlated with adjusted PMBSF area. Although we phenotyped 42 BXD RI strains, the highest number of strains that was detected by the correlation analysis was n = 22.

After searching through the 2000 transcripts, we detected two genes of particular interest, adenylate cyclase 1 (*Adcy1*) and growth associated protein 43 (*Gap43*), that have a significant biological relationship to PMBSF area. The first gene, *Adcy1*, is correlated at r = 0.40 (p = 0.063) with a high mean expression of 16.28. *Adcy1 *is found on chromosome 11 of the mouse starting at 7.08 Mb. The second gene of interest, growth associated protein 43 (*GAP43*), is correlated with total PMBSF area (r = -0.55, p = 0.007) and is found on chromosome 16 starting at 42.18 Mb.

We took advantage of previously published phenotypes by searching for traits that correlated with *Car8*, one of our potential candidate genes. Using the online Allen Brain Atlas [16], we observed that *Car8 *was highly expressed in cerebellar Purkinje cells. *Car8 *has strong negative correlations with volume of CNS regions in BXD RI strains, including basolateral amygdala, hippocampus, cerebellum, and striatum.

## Discussion

### Synopsis

This study was an extension of our previous work [8] in which the total PMBSF/ALBSF area was examined in four common inbred strains and 10 BXD RI strains of mice. In the present study, we collected data from a total of 42 BXD RI strains of mice, two parental strains, and one F1 hybrid. We examined a number of phenotypes including body weight, brain weight, and total PMBSF area in a total of 140 cases. This is the first time that such a wide array of BXD RI strains have been used for mapping a QTL of any cortical area. We found wide continuous variations of total PMBSF area among the BXD RI strains with a 33% difference between highest and lowest values. Using previously established methods of QTL analysis, we identified a suggestive QTL on the proximal arm of chromosome 4 linked to total PMBSF area. This locus contained two interesting candidate genes: *Car8 *and *Rab2*. In addition, correlation analysis with a whole brain microarray expression database revealed a number of highly correlated genes throughout the genome including adenylate cyclase 1 (*Adcy1*), a gene known to disrupt barrel organization of mouse somatosensory cortex [17], and *Gap43*, a gene previously reported to be involved in barrel formation during mouse development [18].

### Technical concerns

One of the most difficult aspects of examining the barrel field in the somatosensory cortex arises from the flattening process of the hemispheres. Only two experimenters performed the cortex flattening process (TJ and CL), with one (TJ) conducting the process in 32/42 (76%) of the BXD RI strains. All reconstructions and measurements were conducted by a single experimenter (TJ) to reduce measurement biases. We also selected the PMBSF to measure since this subfield is the largest, contains well-delineated barrels, and suffers less distortion in the flattening process. This is particularly important, because when the PMBSF and ALBSF were previously examined [8], the ALBSF was often distorted in the flattening process, since the most anterior barrels within the ALBSF were located over a major curvature in the cortex. Therefore, we focused exclusively on the PMBSF in the present study.

In our data set, there was a significant correlation between age and strain, and thus we were unable to test age as a predictor of total PMBSF area in our linear model. However, this should not affect the genetic analysis of total PMBSF area given that the age range in the dataset was fairly compact (40 – 61 days old) and no significant relationship between age and total PMBSF/ALBSF area was previously detected [8].

### Effects of brain and body weights

Brain and body weight measurements correlated significantly. Both also correlated significantly with total PMBSF area. However this correlation was modest at best. This suggests that total PMBSF area is not simply modulated by body and brain weights. In order to dissect these seemingly interrelated phenotypes, we used a linear model that essentially removed the effects of significant predictors of total PMBSF area. This led to the generation of adjusted total PMBSF area values that remove effects of brain weight, the most significant predictor. To gain a visual understanding and further confirm our total PMBSF area QTL, we mapped the raw data of both body and brain weights separately. As evident from body weight and brain weight QTL maps (Figure 2), there were no significant or suggestive QTLs at any locus on the genome. The body weight and brain weight QTL signals that were found on chromosome 4 for both phenotypes do not overlap with the suggestive QTL we detected for total PMBSF area.

### Difference between parental strains

As previously reported [8,19], the average brain weight of C57BL/6J is significantly larger than DBA/2J. It was also previously observed that the DBA/2J mice have a significantly larger ALBSF/PMBSF area than the C57BL/6J mice [8]. In our study, we did not detect an absolute difference between the PMBSF areas of C57BL/6J and DBA/2J when using raw data. Nonetheless, the trend is still present in our data; the DBA/2J PMBSF area is larger than C57BL/6J PMBSF area by approximately 4%. However, after adjusting for brain weight, total PMBSF area of DBA/2J is significantly larger than that of C57BL/6J by approximately 8.5%.

### Total PMBSF area QTL

We report a genome-wide suggestive QTL on the proximal arm of chromosome 4 that is associated with total PMBSF area. Mapping was conducted using total PMBSF area values adjusted for brain weight. As a comparison, raw value total PMBSF area was also mapped. There were no major differences between the two maps with regards to the signal on chromosome 4, however the most notable differences are the reduction of two suggestive QTLs on chromosomes 17 and X when adjusted total PMBSF area values are used. It is worth noting that following bootstrap analysis, we detected that one of our phenotyped strains (BXD73) had a large effect on the significance of our suggestive QTL. When BXD73 was removed, the suggestive QTL on chromosome 4 exceeded the significance level. However, we included BXD73 for completion. Furthermore, the shape of the QTL peak was nearly identical whether BXD73 was included or removed.

Using the marker regression tool in WebQTL, we observed four markers on chromosome 4 that were linked to adjusted total PMBSF area. Interestingly, we previously reported two markers (D8Mit145 and D10Mit3) that were associated with total PMBSF/ALBSF area [8]. However, only 10 BXD RI strains were utilized for mapping in that study. The much larger power of 42 BXD RI strains for the detection of significant QTLs must be highlighted. In addition, different phenotypes were measured, where the previous study measured the entire representation of the whiskers of PMBSF and ALBSF area; here we focused exclusively on the total PMBSF area.

### Candidate genes

Given the small number of genes within the suggestive QTL on chromosome 4, we began exploring the genes that may be responsible for modulating total PMBSF area. In the absence of functional biological data, it is difficult to present a compelling case for any particular candidate gene present within a typically large QTL interval. In this study, we examined prospective genes within a fairly small interval spanning 5.5 to 9.0 Mb on chromosome 4. Two of these genes within this interval, (*Car8 *and *Rab2*), have a high number of SNPs, and particular biological relevance to total PMBSF area.

Carbonic anhydrase-related protein VIII (Car8 or CA-RP VIII) is a *cis*-acting gene that is located within our QTL interval starting at 8.07 Mb and contains 174 SNPs. *Cis*-acting genes are known to regulate their own expression and are considered to be strong candidates for complex traits [20,21]. *Car8 *is a member of the carbonic anhydrase gene family which contains 14 isoforms in humans [22], three of which lack enzymatic activity due to missing one or more of the three zinc-binding histidines critical for catalysis [23]. Those without catalytic activity are known as carbonic anhydrase-related proteins that include *Car8*, *Car10*, and *Car11 *[24]. Murine *Car8*, *Car10*, and *Car11 *have high cDNA and amino acid sequence similarity (89.2–94.5% and 97–100%, respectively) with their human homologues, indicating a fundamental biological role for the carbonic anhydrase-related proteins [25]. *Car8 *is expressed in the cell body of neurons in most parts of the CNS [23]. Early in human gestation, *Car8 *is specifically expressed in neuroprogenitor cells in the subventricular zone and observed in neural cells migrating to the cortex [23].

A more recent study has implicated a role for *Car8 *in motor control in mice [26]. The *Car8 *knockout, known as the *waddles *mutation, is characterized by wobbly side-to-side ataxic movement, typically seen 2 weeks after birth [26]. Thus, *Car8 *appears to be an important gene that is necessary for CNS functioning.

*Rab2 *is also a gene of interest that starts at 8.46 Mb on chromosome 4 under the total PMBSF area QTL. This gene harbors a total of 176 SNPs in the BXD cross and has a *trans*-acing QTL on distal chromosome 1 (LRS > 15), in hippocampus, cerebellum, and whole brain transcriptomes. *Rab2 *belongs to the Rab subfamily of small GTP-binding proteins whose many members are thought to be involved in aiding in the targeting of cytosolic transport vesicles to their many destinations. Perhaps one of the most studied members of this family is the Ras gene. Rabs work with an array of proteins including GTPase Activating Proteins (GAPs) and Guanine Nucleotide Exchange Factors (GEFs) [27]. The Rab2 protein is a resident of pre-Golgi intermediates and is required for protein transport from the endoplasmic reticulum to the Golgi complex and is essential for the maturation of pre-Golgi complexes [28].

### Correlated gene expression profiles

Whole-brain mRNA expression profile correlation analysis revealed two genes that correlate significantly with total PMBSF area. The first, adenylate cyclase I (*Adcy1*), is a neurospecific membrane bound protein that catalyzes the formation of the crucial second-messenger cAMP [17]. Expression of *Adcy1 *is known to be linked with regions of the brain associated with plasticity, including the hippocampus and cerebral cortex [29]. Mice with knockout *Adcy1 *gene (barrelless mutation) do not develop the commonly observed barrel fields in the SI cortex [17]. However, such mice are still known to have normal topological organization of the SI cortex [30]. In addition to showing that disrupted *Adcy1 *displays a barrelless phenotype, for the first time, a second messenger was directly linked to cortical development.

A second gene of interest, *Gap43*, negatively correlated with total PMBSF area (r = -0.52, p = 0.02). Although not highly expressed, we report it for completion and more importantly, for its significant biological role. GAP43 expression following stroke is strikingly similar to staining of GAP43 in first and second postnatal weeks in the barrel cortex (Erzurumlu et al., 1990). GAP43 is shown to be involved in post-stroke axonal sprouting [31] and is important in critical stages of neurodevelopment of the barrel cortex [18]. Erzurumlu and colleagues also showed that *Gap43 *expression pattern was similar to the barrel field pattern, including the PMBSF region [18]. They reported the transient nature of GAP43, since almost no expression of GAP43 was observed after post-natal day 7.

## Conclusion

Using 42 BXD RI strains, we identified a novel suggestive QTL on chromosome 4 that is associated with total PMBSF area. Within this QTL, we propose that *Car8 *is a likely candidate gene given that it has a strong *cis*-QTL with multiple independent probes and plays an essential role in CNS functioning. Taken together, our findings are a further step toward the identification of candidate genes that may be responsible for the modulation of total barrel subfield size in the SI cortex.

## Methods

### Animals

A total of 140 mice from 45 strains was used in this study. Phenotypic data were collected from 122 mice from 42 BXD RI strains (n = 2.90 ± 0.22 per strain) ranging between 40 to 61 days of age (average 46.66 ± 0.44 days). All but six strains, BXD 55 (F18), BXD 56 (F14), BXD 71 (F16, F17), BXD 80 (F19), BXD 83 (F17), and BXD 84 (F17), had been inbred for at least 20 generations. The phenotypes of the two parental inbred strains (C57BL/6J, DBA/2J) and one F1 strain (B6D2F1) were also analyzed. A total of 13 animals was phenotyped from the parental strains and 5 animals were phenotyped from the F1 strain. The age range of these mice was similar to that of the BXD strains (average 45.06 ± 0.64 days). While all strains were not balanced evenly with respect to sex, our data contained at least one male and one female from 38 of the total 44 strains phenotyped.

All animals were maintained at a temperature of 22°C on a 12 hr light/dark cycle with 35–40% humidity in a specific pathogen-free (SPF) environment at the University of Tennessee Health Science Center Animal Facility. Animals were fed 5% fat Agway Prolab 3000 rat and mouse chow. All experimental procedures were carried out in accordance with *Principles of Laboratory Animal Care *(NIH publication No. 86–23, revised 1985) and were approved by the Institutional Animal Care and Use Committee at the University of Tennessee Health Science Center. The Animal Care Facility is AAALAC approved.

### Tissue preparation

Tissue preparation was conducted essentially as previously described [8]. Mice were deeply anesthetized with Nembutal (50 mg/kg) and perfused intracardially with 0.9% saline solution followed by 4% paraformaldehyde in 0.3 M sodium phosphate-buffered saline (NaPBS) at a pH of 7.4. The brain was removed from the skull, blocked, hemispheres separated and flattened between two Plexiglas plates, and flattened hemispheres were refrigerated overnight in 4% paraformaldehyde. Body weight and brain weight (with and without cerebellum and olfactory bulbs, cerebellum and brainstem weight, olfactory bulb, and left and right cortices) were measured prior to flattening. The following day, hemispheres were sectioned at 100 μm-thickness using a Vibratome and sections were placed in test tubes containing 0.01 M potassium phosphate-buffered saline (KPBS). Sections were washed in KPBS (3 × 10 min), stained with cytochrome oxidase (CO) to visualize the PMBSF, and mounted on gelatin-coated slides. Slides were left to air dry before they were coverslipped with Permount.

### PMBSF reconstruction

**Figure 4:**
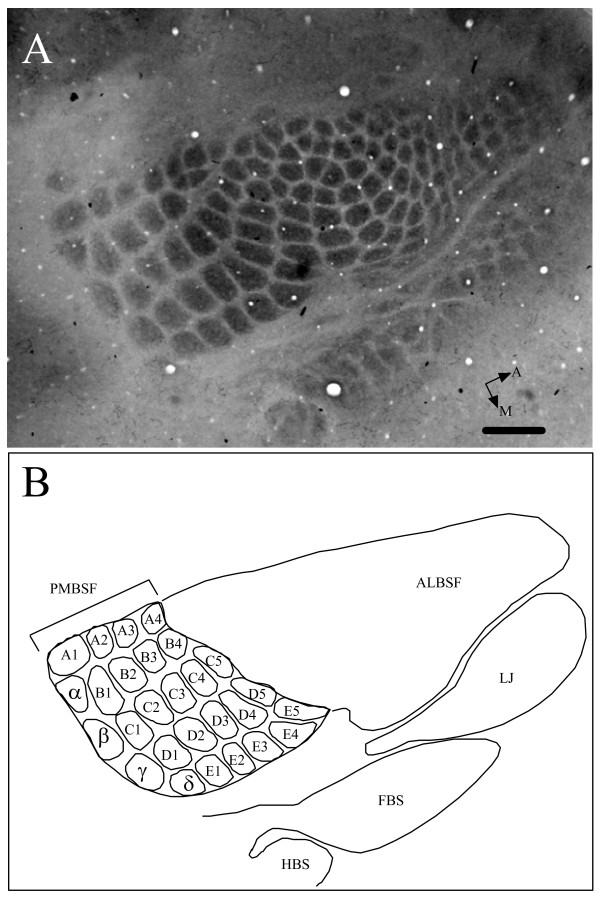
Example of a CO stained barrel field in an RI (BXD63) mouse. (A) Photomicrograph of the entire barrel field. (B) Line drawing showing prominent barrel subfields: *PMBSF *posterior medial barrel subfield, *ALBSF *anterior lateral barrel subfield, *LJ *lower jaw, *FBS *forepaw barrel subfield, and *HBS *hindpaw barrel subfield. Individual PMBSF barrels are outlined and labeled. Five prominent rows of barrels (A through E) are seen. In addition, four posteriorly located straddler barrels (α, β, γ, δ) are also seen. Total area of PMBSF (barrels and septal regions between barrels) was measured as outlined in figure. Scale 500 μm.

Slides containing PMBSF barrels were viewed under a light microscope (Nikon Optiphot) and photographed with a digital camera (Kodak Coolpix 9000). Photomicrographs containing the PMBSF were transferred and stored on a Macintosh (G5) computer, and the PMBSF reconstructed using Adobe Photoshop CS. In cases where the entire PMBSF did not appear on a single section, blood vessels were used as fiducials to align adjacent sections to produce a composite reconstruction. The total PMBSF was measured using NIH program ImageJ 1.33 u. The PMBSF typically consisted of four straddler barrels (α, β, γ, δ), the first four barrels of rows A and B, and the first five barrels of rows C through E, resulting in a total of 27 barrels. The total PMBSF area was defined by drawing a line around the 27 barrels to produce an outline that was measured using ImageJ. Figure 4 shows a section containing the entire barrel field. All reconstructions and measurements were done by a single experimenter (TJ) to minimize technical error. Measurements were stored in an Excel spreadsheet for subsequent analysis.

### Left and right side asymmetry

Left and right PMBSF areas of all mice were measured and compared against one another as a possible gauge of technical error; our assumption being that R-L asymmetry would be modest compared to technical sources of error. A paired t-test and Pearson's product-moment correlation were used to compare the total PMBSF area for each of the two hemispheres. No significant differences were observed between total PMBSF area of hemispheres (p > 0.60) and right and left PMBSF areas showed a high degree of correlation (r > 0.85). Therefore, we averaged the PMBSF areas for both hemispheres in subsequent analyses.

### Statistical analysis and data modeling

All basic statistical analyses and tests were carried out using Data Desk (6.1) or Excel. Standard error of means was calculated by dividing the standard deviations by the square root of the number of samples.

We modeled our data based on careful correlation examinations to determine what factors may be used as predictors of PMBSF area. Correlation significance was determined using simple regression p-values. A general linear model was utilized to determine predictors of PMBSF area. Age, sex, body weight, and brain weight were all considered as possible predictors of PMBSF area. The linear model (Data Desk 6.1) that was initially used contained all possible predictors of total PMBSF area. Non-significant predictors were then removed in a sequential manner until only significant predictors were left.

### Heritability

Broad-sense heritability (h^2^) was measured by comparing between-strain and total variances, using the Hegmann and Possidente method where h^2 ^= V_A_/[V_A _+ 2V_E_] (V_A _= genetic variance and V_E _= environmental variance) [32]. Broad-sense heritability provides an estimate of the total variance due to genetic factors.

### QTL mapping

QTL analysis involves classifying strains based on their genotypes at discrete chromosomal markers and comparing these groups with a quantitative variable, total PMBSF area in the present study. If the variation in phenotype matches the differences in genotype, then a gene locus will be detected [3].

Raw and adjusted PMBSF data from BXD RI strains were used to map potential QTLs. QTL maps of brain and body weights were also generated. All QTL maps were generated using the online WebQTL [33]. Markers are reported with genome-wide significance and those considered suggestive based on 1000 permutation tests. It is important to note, that a slight variability in significant and suggestive thresholds will be detected each time the maps are recomputed because of the random generation of the permutations. Three types of QTL mapping regressions were utilized in this study and they include: simple regression, interval regression, and marker regression. The likelihood ratio statistic (LRS) was used to assess genome-wide significance in linkage analysis. Logarithm of odds (LOD) values are obtained simply by dividing the LRS by 4.6.

### Correlative analysis

GeneNetwork provides users with an array of analytical tools to compare a given trait with a number of data sets available from other experimenters. Microarray data of gene expression in the brain and data of other phenotypes are two such examples of possible tools. For this study, we correlated our PMBSF area phenotype with the mRNA expression level data "INIA Brain mRNA M430 (Jan 06) PDNN." Pearson's product-moment correlations were utilized. Unfortunately, we could not take advantage of the trait-to-trait correlations for this study given that many of the phenotyped strains here have not been available for a long period of time, thus experimenters have not yet collected a plethora of phenotypes for comparison. However, we did take advantage of other published phenotypes by correlating them with potential candidate genes, specifically, *Car8*.

## Authors' contributions

TAJ carried out the animal procedures including tissue processing, brain measurements, analysed the data, and drafted the manuscript. LL analysed the data and conducted data modeling, CXL carried out animal procedures, including tissue processing and brain measurement and assisted with data analysis, RWW analysed data and helped with manuscript preparation, RSW conceived of and coordinated the study, and drafted the manuscript. All authors read and approved the final manuscript.

## References

[B1] Williams RW, Morrison J, Hof P (1998). Neuroscience meets quantitative genetics: using morphometric data to map genes that modulate CNS architecture. The 1998 Short Course in Quantitative Neuroanatomy.

[B2] Zygourakis CC, Rosen GD (2003). Quantitative trait loci modulate ventricular size in the mouse brain. J Comp Neurol.

[B3] Lu L, Airey DC, Williams RW (2001). Complex trait analysis of the hippocampus: mapping and biometric analysis of two novel gene loci with specific effects on hippocampal structure in mice. J Neurosci.

[B4] Peirce JL, Chesler EJ, Williams RW, Lu L (2003). Genetic architecture of the mouse hippocampus: identification of gene loci with selective regional effects. Genes Brain Behav.

[B5] Airey DC, Lu L, Williams RW (2001). Genetic control of the mouse cerebellum: identification of quantitative trait loci modulating size and architecture. J Neurosci.

[B6] Airey DC, Wu F, Guan M, Collins CE (2006). Geometric morphometrics defines shape differences in the cortical area map of C57BL/6J and DBA/2J inbred mice. BMC Neurosci.

[B7] Beatty J, Laughlin RE (2006). Genomic regulation of natural variation in cortical and noncortical brain volume. BMC Neurosci.

[B8] Li CX, Wei X, Lu L, Peirce JL, Williams RW, Waters RS (2005). Genetic analysis of barrel field size in the first somatosensory area (SI) in inbred and recombinant inbred strains of mice. Somatosens Mot Res.

[B9] Woolsey TA, Van der Loos H (1970). The structural organization of layer IV in the somatosensory region (SI) of mouse cerebral cortex. The description of a cortical field composed of discrete cytoarchitectonic units. Brain Res.

[B10] Welker C, Woolsey TA (1974). Structure of layer IV in the somatosensory neocortex of the rat: description and comparison with the mouse. J Comp Neurol.

[B11] Dawson DR, Killackey HP (1987). The organization and mutability of the forepaw and hindpaw representations in the somatosensory cortex of the neonatal rat. J Comp Neurol.

[B12] Pearson PP, Oladehin A, Li CX, Johnson EF, Weeden AM, Daniel CH, Waters RS (1996). Relationship between representation of hindpaw and hindpaw barrel subfield (HBS) in layer IV of rat somatosensory cortex. Neuroreport.

[B13] Waters RS, Li CX, McCandlish CA (1995). Relationship between the organization of the forepaw barrel subfield and the representation of the forepaw in layer IV of rat somatosensory cortex. Exp Brain Res.

[B14] Welker C (1976). Receptive fields of barrels in the somatosensory neocortex of the rat. J Comp Neurol.

[B15] Williams RW, Gu J, Qi S, Lu L (2001). The genetic structure of recombinant inbred mice: high-resolution consensus maps for complex trait analysis. Genome Biol.

[B16] Lein ES, Hawrylycz MJ, Ao N, Ayres M, Bensinger A, Bernard A, Boe AF, Boguski MS, Brockway KS, Byrnes EJ, Chen L, Chen L, Chen TM, Chin MC, Chong J, Crook BE, Czaplinska A, Dang CN, Datta S, Dee NR, Desaki AL, Desta T, Diep E, Dolbeare TA, Donelan MJ, Dong HW, Dougherty JG, Duncan BJ, Ebbert AJ, Eichele G, Estin LK, Faber C, Facer BA, Fields R, Fischer SR, Fliss TP, Frensley C, Gates SN, Glattfelder KJ, Halverson KR, Hart MR, Hohmann JG, Howell MP, Jeung DP, Johnson RA, Karr PT, Kawal R, Kidney JM, Knapik RH, Kuan CL, Lake JH, Laramee AR, Larsen KD, Lau C, Lemon TA, Liang AJ, Liu Y, Luong LT, Michaels J, Morgan JJ, Morgan RJ, Mortrud MT, Mosqueda NF, Ng LL, Ng R, Orta GJ, Overly CC, Pak TH, Parry SE, Pathak SD, Pearson OC, Puchalski RB, Riley ZL, Rockett HR, Rowland SA, Royall JJ, Ruiz MJ, Sarno NR, Schaffnit K, Shapovalova NV, Sivisay T, Slaughterbeck CR, Smith SC, Smith KA, Smith BI, Sodt AJ, Stewart NN, Stumpf KR, Sunkin SM, Sutram M, Tam A, Teemer CD, Thaller C, Thompson CL, Varnam LR, Visel A, Whitlock RM, Wohnoutka PE, Wolkey CK, Wong VY, Wood M, Yaylaoglu MB, Young RC, Youngstrom BL, Yuan XF, Zhang B, Zwingman TA, Jones AR (2007). Genome-wide atlas of gene expression in the adult mouse brain. Nature.

[B17] Abdel-Majid RM, Leong WL, Schalkwyk LC, Smallman DS, Wong ST, Storm DR, Fine A, Dobson MJ, Guernsey DL, Neumann PE (1998). Loss of adenylyl cyclase I activity disrupts patterning of mouse somatosensory cortex. Nat Genet.

[B18] Erzurumlu RS, Jhaveri S, Benowitz LI (1990). Transient patterns of GAP-43 expression during the formation of barrels in the rat somatosensory cortex. J Comp Neurol.

[B19] Airey DC, Robbins AI, Enzinger KM, Wu F, Collins CE (2005). Variation in the cortical area map of C57BL/6J and DBA/2J inbred mice predicts strain identity. BMC Neurosci.

[B20] Chesler EJ, Wang J, Lu L, Qu Y, Manly KF, Williams RW (2003). Genetic correlates of gene expression in recombinant inbred strains: a relational model system to explore neurobehavioral phenotypes. Neuroinformatics.

[B21] Kempermann G, Chesler EJ, Lu L, Williams RW, Gage FH (2006). Natural variation and genetic covariance in adult hippocampal neurogenesis. Proc Natl Acad Sci U S A.

[B22] Hewett-Emmett D (2000). Evolution and distribution of the carbonic anhydrase gene families. Exs.

[B23] Taniuchi K, Nishimori I, Takeuchi T, Fujikawa-Adachi K, Ohtsuki Y, Onishi S (2002). Developmental expression of carbonic anhydrase-related proteins VIII, X, and XI in the human brain. Neuroscience.

[B24] Tashian RE, Hewett-Emmett D, Carter N, Bergenhem NC (2000). Carbonic anhydrase (CA)-related proteins (CA-RPs), and transmembrane proteins with CA or CA-RP domains. Exs.

[B25] Taniuchi K, Nishimori I, Takeuchi T, Ohtsuki Y, Onishi S (2002). cDNA cloning and developmental expression of murine carbonic anhydrase-related proteins VIII, X, and XI. Brain Res Mol Brain Res.

[B26] Jiao Y, Yan J, Zhao Y, Donahue LR, Beamer WG, Li X, Roe BA, Ledoux MS, Gu W (2005). Carbonic anhydrase-related protein VIII deficiency is associated with a distinctive lifelong gait disorder in waddles mice. Genetics.

[B27] Armstrong J (2000). How do Rab proteins function in membrane traffic?. Int J Biochem Cell Biol.

[B28] Tisdale EJ, Balch WE (1996). Rab2 is essential for the maturation of pre-Golgi intermediates. J Biol Chem.

[B29] Choi EJ, Xia Z, Villacres EC, Storm DR (1993). The regulatory diversity of the mammalian adenylyl cyclases. Curr Opin Cell Biol.

[B30] Welker E, Armstrong-James M, Bronchti G, Ourednik W, Gheorghita-Baechler F, Dubois R, Guernsey DL, Van der Loos H, Neumann PE (1996). Altered sensory processing in the somatosensory cortex of the mouse mutant barrelless. Science.

[B31] Carmichael ST, Archibeque I, Luke L, Nolan T, Momiy J, Li S (2005). Growth-associated gene expression after stroke: evidence for a growth-promoting region in peri-infarct cortex. Exp Neurol.

[B32] Hegmann JP, Possidente B (1981). Estimating genetic correlations from inbred strains. Behav Genet.

[B33] WebQTL http://www.genenetwork.org.

